# Inflammation and Ulceration of the Cervix Uteri, Its Pathology and Treatment

**Published:** 1858-01

**Authors:** V. H. Taliaferro

**Affiliations:** Atlanta, Geo.


Medical and Surgical Journal.

VOL. III.] JANUARY, 1858. [No. V.

### ORIGINAL COMMUNICATIONS.

### ARTICLE I.

*Inflammation and Ulceration of the Cervix Uteri, its Pathology and Treatment.* By V. H. TALIAFERRO, M. D., Atlanta, Geo.

In the present article, it will somewhat facilitate our purpose to notice very briefly some of the anatomical peculiarities of the cervix uteri. The body of the uterus is much more dense and firm than the cervix, from the fact of the absence in the body, of cellular structure, while the neck is rendered soft and elastic by its presence. With this difference, they are alike composed of muscular and mucus tissue. The body of the uterus is sparsely supplied with blood vessels from the smaller branches of the ovarian and uterine arteries, while the neck is abundantly supplied from the larger branches of the same vessels. Thus we see from the greater vascularity of the cervix, the cause of its more frequent liability to disease.

The cervix uteri is freely supplied with nerves from the hypogastric plexuses, spermatic and aortic, which explains its sympathetic relations with various parts of the system, and also its obtunded sensibility to local impressions, its nervous communication being almost exclusively sympathetic.

The mucus membrane which lines the os and canal of the cervix, is thickly studded with mucus follicles.

Now, we at once perceive, from our very brief notice of the anatomical structure of the cervix uteri, its very high degree of organization, and consequently, its susceptibility to disease. The os, in its normal condition, is firmly closed, not admitting very frequently the smallest size probe, while the cervix is smooth and velvety in appearance, its color pale pink, and covered with a slight mucus secretion. To the touch, it is soft and elastic, changing readily its position from pressure. When diseased, the firmly closed os becomes dilated ; the smooth and beautiful pink colored cervix, is changed to livid red from inflammatory action, darkened from congestion, or roughened by ulceration.

Congestion of the cervix uteri supervenes upon the approach of each menstrual period, which subsides with the healthy cessation of the catamenia, leaving the cervix in its normal condition; but if there be a sudden arrest of the menstrual flow, to which the female is subject from a great variety of causes, we can very readily perceive its liability to take on diseased action, when we remember that congestion invariably precedes inflammatory action, and that although this condition of the cervix is necessary for the establishment of the physiological function of the uterus, it is that very condition which, if interfered with, will very probably establish a diseased process in the organ itself.

Not unfrequently does the female date her first uterine disturbance to the wetting of her feet, the use of cold ablutions, or to some imprudence while menstruating, and we are never surprised that such is the result, for we could not expect a more favorable result than inflammatory action, from the sudden arrest of a secretion from so highly vitalized an organ, consisting as it does, of a net work of blood vessels, and nerves, interwoven into muscular, mucus, and cellular structures.

Under these circumstances, it is a matter of no little surprise that men high in medical authority, should deny the existence of inflammation or ulceration of the cervix uteri, unless as a rare occurrence from extraordinary circumstances.

Notwithstanding the uterus is endowed with a peculiar physiological function, it is not unlike other organs, endowed with the capacity of resisting morbid action. The position of the cervix uteri in the upper extremity, or "cul de sac" of the vagina; its susceptibility during menstrual periods; its highly organized structure; the danger of its abrasion, rupture, &c., during parturition; its excitement from sexual connection, will all bear us out in the affirmation, that the cervix uteri is the subject of very frequent disease.

In the virgin, menstrual derangement is by far the most frequent cause of uterine disease, but with the married female, a variety of causes occur to interfere with the normal conditions of the uterus.

Parturition, as would naturally be inferred, is a frequent source of inflammatory, or ulcerative disease of the cervix uteri.

The violent throes of labor upon the undilated os seems well calculated to lacerate and contuse its delicate lining membrane; and when such a result does occur, the recuperative forces of the system may restore the organ to its normal condition, but if there should exist at the same time constitutional debility, attended with loss of blood, retention of portions of the placenta, or other debilitating causes, an exhausting leucorrhœa will most usually supervene, attended with its host of ailments, such as hysteria, debility, dyspepsia, hepatic disturbance, urinary irritation, constipation, diarrhœa, cephalalgia, palpitation, dysmenorrhœa, menorrhagia, &c.

Sexual connection, when carried to excess, is not unfrequently the source of diseased cervix uteri, especially when there exists an undue susceptibility of the uterine system.

Syphilitic, or gonorrhœal inflammation may extend itself from the vaginal canal to the cervix of the uterus, and produce disease of specific character there.

Now, we can very readily perceive from the variety of opposite causes, which produce uterine disease, the vast importance in the application of treatment, to ascertain the true pathological condition of our patient, and remove, if possible, the original cause of the morbid action.

When disease of the cervix does occur many circumstances operate against the successful employment of treatment, and in consequence, a considerable length of time must be allowed to heal a simple ulcer or slight abrasion.

The position and anatomical stucture of the cervix, with the excitement attendant upon each menstrual period, present important barriers, to the speedy relief of its disease.

Any cause which produces disturbance of the functions of the uterus may produce disease in its structure, and we are well satisfied that a most frequent cause of disturbed menstruation, is constitutional debility. Uterine disease most frequently occurs in females of delicate and debilitated constitutions, and it is no doubt from the fact that with them the menstrual function of the uterus is almost invariably disturbed in such cases. We remember very rarely to have seen chronic inflammatory, or ulcerative disease of the cervix uteri in a female of robust healthy constitution. With these facts it would appear that the state of the constitution is of no minor importance in the treatment of this class of diseases. Whether constitutional debility is the effect or the cause of uterine disease it should demand our most careful attention. The symptoms which lead us to suspect disease of the cervix uteri are both constitutional and local, the most important only of which we shall notice.

As a consequence of the intimate connection of the uterus with the sympathetic nervous system, the disturbance which supervenes upon its disease extends to most of the organs of the system.

The most frequent seat of pain is in the region of the ovaria, in the back and hips, sometimes coursing the lower extremities. Hepatic derangement is not unusual, and may lead the unguarded practitioner to suspect organic disease of the liver. We have seen chronic ulceration of the cervix mistaken and treated for "liver disease."

The disturbance of the gastric function is of frequent occurrence, and may be of such character as very materially to interfere with the assimilative process, and we may have as a result of this disturbance, general debility and anæmia, attended with excessive irritability of the nervous system.

If this state of things is unregarded, our patient may die, not from the extent of the primary disease, but from an extreme state of exhaustion and anæmia, consequent upon continued nervous irritability, and deficient assimilation.

The lungs, the heart, the liver, the stomach, the kidneys, the bladder, are all the subject of derangement from diseased uterus. One symptom, which we look upon as important, and not noticed by standard authors upon uterine diseases, is spasmodic contraction of the abdominal muscles, as an effect of spinal irritation, which is not unfrequently produced by disease of the cervix of the uterus.

The only mention we remember to have seen of this condition of the abdominal muscles, was in an article published in the London Lancet, by E. Headlam Greenhow, M. D., Lecturer at St. Thomas' Hospital, etc., and by several journals republished, upon "Phantom Tumors," in which several cases are reported of apparently distinct tumors, which were ascertained to be muscular contraction, kept up by the irritation of the spine, produced by the uterine disease. These tumors were dispersed upon the removal of the cause.

We have had an opportunity of witnessing a tumor of this character, in which the patient would not be convinced, but that it was a growth within the abdomen of very serious character, notwithstanding that it frequently changed its position was moveable upon pressure, and sometimes altogether absent.

As to the true nature of this tumor we were for a considerable time uncertain, but from its increased enlargement and disturbance upon the approach of the menstrual period, we were satisfied of its connection in some way, with the uterine disease which was present. These tumors are very firm upon pressure, do not increase the measurement of the abdomen, and produce an unpleasant drawing sensation of the part, and are dispersed by relief of the spinal irritation, which has produced them. The writer of the article referred to says, "In considering the history of the cases I have described, it is noticeable that all of them were females, suffering from some disturbance of the uterine function; and that whilst spinal irritation unequivocally existed in three of the patients, its presence may not unfairly be inferred in both the others.

"The real nature of these tumors is spasmodic; their seat probably the abdominal muscles; for although in every instance I have seen, the tumor appeared to be in the abdominal cavity, the melting away of my last case under manipulation, is inconsistent with the belief that they are very deeply seated. The cause is spinal irritation, the irritated spinal nerves producing spasm in the muscles to which they are distributed."

Leucorrhœa is a most frequent attendant upon uterine disease, and depends in quantity and quality upon the extent and character of the malady.

Other symptoms than these may present themselves, but as before remarked, it has been our object to notice only the most important.

From the symptoms and conditions which we have enumerated, we will be led to suspect uterine disease; but the true pathological condition of the uterus can only be ascertained by the aid of the speculum exposing fully to view the cervix. An educated touch will do much in determining the nature of the disease, but can only be auxiliary to the use of the speculum.

When the cervix is brought to view, we at once determine whether there be a departure from its normal condition; and whether there be inflammation, ulceration, abrasion, induration or hypertrophy; its true pathological condition being determined, we have done much towards the accomplishment of our purpose, viz: a cure.

The treatment will, of course, be both constitutional and local, governed, in a great degree, by the character, extent and duration of the disease. The importance of the condition of the constitution of the female laboring under uterine disease, cannot be too highly regarded, provided we are not led to extreme, or exclusive views. Since the importance of local measures for uterine diseases have been fully established, we are too prone to entirely disregard the condition of our patients' constitution, considering that all derangement, no matter how severe, is superinduced by the local disease, to which alone the application of treatment must be directed. In speaking of the causes of disease of the uterus, it will be remembered, that allusion was made to constitutional debility, as a frequent cause of derangement of *the function* of the organ, and as a result of that derangement, inflammatory, or ulcerative disease of the organ itself. When such a cause does exist, the importance of treatment directed to the condition of the constitution, can not be too highly estimated.

Under such circumstances, let us ascertain the remote cause of the disturbance, and if possible, remove it, thereby relieving the general system of its direct and injurious influence, and the uterus of its indirect and hazardous effects. With such a cause existing, do we not at once perceive, that the entire cervix uteri might be consumed by caustics, and still the disease would persist, as a result of the continuance of its original cause.

It will, no doubt, be denied that inflammatory or ulcerative disease of the cervix uteri ever occurs as a result of constitutional debility; but when we remember, that the uterus becomes, monthly, the seat of congestion, and of morbid congestion, unless relieved by an undisturbed performance of its physiological function, we are not surprised at the occurrence of inflammatory action, though it may be, not sthenic, but of such character as that occurring in the lungs in Typhoid fever, as a result of congestion; that congestion being, in our opinion, caused, principally, by the extreme debility which attends the disease in such cases.

As to the greater importance of *either* local or constitutional treatment, we have only to say, that the cause which has produced the disease, the condition, &c., of the patient, both locally and constitutionally, will readily determine the preference of either one or the other, or both plans of treatment.

In very delicate and susceptible nervous temperaments, very alarming prostration may ensue as a result of slight uterine disease, and in such cases, it is very readily perceived, that our efforts should be mainly directed to the restoration of the fast failing powers of life, to the building up of the recuperative forces of the system, and thereby be enabled to call to our assistance all the powers of the "vis medicatrix naturæ," in throwing off from the system the morbid action. We may commence the treatment of disease of the cervix, by exclusively local treatment, supposing the condition and strength of the constitution to be such as not to demand attention; but we should remember that the derangement of the assimilative process, with the continued nervous irritability, which almost invariably attends the disease, is, by degrees, though slow it be, doing its work upon the constitution.

Support, then, to the general system, is of no minor importance in the treatment of this class of diseases. Tone to the digestive aparatus, a healthy assimilative process, should, as far as possible, be maintained by the use of proper diet, tonics, and such means as tend to promote digestion, and give strength and tone to the constitution.

When the strength will admit, the proper kind, and sufficient amount, of exercise should be enjoined as an important feature in the treatment, for upon it will depend to no inconsiderable extent the happy termination of the disease.

The patient should confine herself to bed only when her failing strength compels her, or during the aggravation of her disease consequent upon the excitement of the menstrual periods.

Moderate and frequent exercise in the open air will be beneficial, not only physically, but mentally, by diverting the mind from the nature and seat of disease.

Alteratives, such as Iodide of Iron, Iodide of Mercury, Iodide of Potassium, &c., will, when indicated, be beneficial; but we have less confidence in the internal administration of medicines, than in exercise, diet, bathing, friction to the skin, &c., unless the urgency of symptoms demand, for a time, exclusive attention.

Yet, while we have given so much importance to the treatment and condition of the constitution in uterine diseases, we consider local applications to the cervix itself as vastly essential, for the speedy and permanent establishment of cure.

In inflammation of the neck of the uterus, the application of leeches and counter irritation, with a frequent use of sol. of nitrate of silver will usually suffice; but, if there be in conjunction an ulcer, it should be touched with the solid nitras argenti.

This condition of the neck of the uterus is frequently attended with some degree of hypertrophy or induration, when the application of caustics will be less efficient than Iodine or Mercury; or what is preferable, blistering the cervix as recommended by Dr. Johns, ex-assistant to the Dublin Lying in Hospital, &c. He uses the concentrated sol. of cantharides in sulphuric ether, mixed with the ordinary sol. of gutta percha in chloroform, in proportion of two parts of the former to one of the latter.

We have used this preparation, and find it to be a most efficient application, when the ulceration is attended with any undue congestion or induration.

It will be necessary in the treatment of these diseases, to use in conjunction with the other treatment, vaginal injections, in order to prevent an accumulation of the discharge in the vagina, and about the cervix. Where tenderness and irritability exist, the injection should be of a soothing character.

While we would like to have prolonged our remarks upon this part of the subject, we feel that we have already trespassed upon the space allowed us in the pages of this journal.

* Half-yearly Abstract of the Medical Sciences.

